# Anxiety, anhedonia, and related food consumption in Israelis populations:An online cross-sectional study two years since the outbreak of COVID-19^☆^

**DOI:** 10.1016/j.heliyon.2023.e17211

**Published:** 2023-06-15

**Authors:** E. Fleischer, L. Landaeta-Díaz, G. González-Medina, O. Horovitz

**Affiliations:** a The Physiology and Behavior Laboratory, Tel-Hai Academic College, 9977 North Districts, Israel; b Psychology Department, Tel-Hai Academic College, 9977 North Districts, Israel; c Faculty of Health and Social Sciences, University of the Americas, Av. Walker Martínez 1360, piso 3, Edificio A, La Florida, Santiago, Chile; d Faculty of Medicine, Department of Public Health, Pontifical Catholic University of Chile, Av. Libertador Bernardo O'Higgins 340, Santiago, 8331150, Santiago, Chile

**Keywords:** COVID-19, Anxiety, Anhedonia, Food consumption, Body-weight

## Abstract

**Background:**

The COVID-19 pandemic has significantly impacted daily life. Beyond severe health and economic consequences, psychological consequences have surfaced that require in-depth research to understand the pandemic's effects on mental health. Aims: This study aimed to evaluate the association between anxiety levels and anhedonia with food consumption patterns and changes in body weight over the two years since the COVID-19 outbreak in Israel.

**Methods:**

This cross-sectional study utilized non-randomized sampling through an online survey that included 741 study participants aged 18 to 94. participants were asked to complete the Beck's Anxiety Questionnaire, the Snaith-Hamilton Pleasure Scale for Anhedonia Measurement, the Mediterranean Nutrition Questionnaire, and self-reports of body weight and serving size changes.

**Results:**

Those who reported severe anxiety and anhedonia reported the highest intake of fats, sugars, and carbohydrates and the highest weight gain (e.g., Butter and cream food: severe anxiety (M = 1.342, SEM = 0.217); low anxiety (M = 0.682, SEM = 0.042), Sweet pastries: severe anxiety (M = 4.078, SEM = 0.451); low anxiety (M = 3.175, SEM = 0.436)). Anhedonic participants consumed more sweetened beverages (M = 0.987, SEM = 0.013) than hedonic participants (M = 0.472, SEM = 0.231). Among participants that gained weight, severe anxiety participants consumed significantly more salty pastries (M = 2.263, SEM = 0.550) than those with low anxiety (M = 1.096, SEM = 0.107; p = .003). A significant interaction was found between weight, anxiety, and consuming salty pastries. High anxiety subjects and weight gain declared the highest intake of this food (p = .018); Significant interactions were found between those with severe anxiety and anhedonia, who reported the highest consumption of butter and cream (p = .005) and salty pastries (p = .021). Significant associations were found between weight and anhedonia and weight and anxiety levels (p = .000, p = .006 – respectively).

**Conclusions:**

The outbreak of COVID-19 and its long-term presence strengthen the negative psychological aspects and increase the consumption of foods high in fat and sugar. Further attention to nutritional health is needed since crises may occur, and we must be prepared to prevent adverse consequences.

## 1 Introduction

In December 2019, Coronavirus disease 2019 (COVID-19), a highly infectious disease caused by the SARS-CoV-2 virus, has spread rapidly since its first outbreak in Wuhan, China [1]. Soon after, the World Health Organization (WHO) identified COVID-19 as a pandemic. By 2020, the U.S. Food and Drug Administration (FDA) had declared an international emergency [2]. Thus far, it has resulted in the deaths of 6.45 million people and the morbidity of 596 million sick and recovered people worldwide. Despite the development of vaccines, new variants continue to appear, causing an increase in the coefficient of infection in many countries [3].

The pandemic context has brought the need for young and old to cope with psychological stress and uncertainty [4]. Previous studies have suggested that public health emergencies can significantly impact the individual's psychological and mental state, expressed in anxiety (i.e., excessive worry and apprehensive expectations about several events or activities, [5]), depression, stress, loneliness, fear, and confusion [6]. This was further stressed in a recent meta-analysis pointing out that COVID-19 causes physical health concerns and results in several psychological disorders, specifically anxiety and depression [4].

An epidemic usually occurs in waves, meaning the infection coefficient alternately rises and falls. This situation perpetuates by a lack of control, persistent fear, instability in routine, inability to plan for the long term**,** and maintaining a high**-**stress level over time. These have long-term physical and mental consequences even after eradicating the pandemic [7].

Fear and anxiety are associated with little desire and motivation to eat out of a sense of pleasure [8]. The restrictions and social distancing were pervasive during the COVID pandemic creating changes in eating habits and the frequency of physical activity, which subsequently affected body weight, health, and quality of life [9]. Those restrictions of movement and prolonged stays at home encouraged increased consumption of **“**tasty**”** food and snacks and food ordering from restaurants [10]. This aligns with studies pointing out that people's eating behavior was altered during the COVID-19 pandemic [11,12]. In situations of anxiety or negative stress, people prefer to consume hedonically rewarding foods, that is, foods high in sugar and fat [13]. Since the outbreak of the COVID-19 pandemic, there has been an increase in the consumption of fast foods, snacks, chocolate, fried and processed foods, and traditional comfort foods. These foods affect the quality of one's diet and the maintenance of body health and cause weight gain [14,15]. A study conducted in Israel found an association between anxiety, diet quality, and body weight. More than a third of the participants reported that their diet before COVID-19 was healthier than the one they currently adhere to Ref. [14]. Landaeta-Díaz et al. (2021) studied the variables of anxiety, anhedonia (which refers to deficits in the capacity to feel pleasure and take an interest in things [5]), food consumption, and weight during the COVID-19 pandemic in Chile. Their findings indicated an increase in food consumption, food ratios, and body weight, all of which were associated with an increase in the level of anxiety. Anhedonia, however, was found to be associated only with increased body weight. The increased consumption of foods high in sugar and fats (such as fried foods, snacks, and sweets) seen in that study was also associated with increased body weight. The study indicates the severe psychological consequences of the COVID-19 pandemic on Chilean citizens' physical and mental health [15].

Nevertheless, there is insufficient evidence of the potential association between psychological symptoms and food patterns two years after the pandemic outbreak. Indeed the literature about the relationship between mental health and food consumption is mainly based on opinions not scientifically grounded [16].

Understanding and expanding knowledge about the indirect of another health problem during the pandemic will be an essential basis for dealing with other global crises. Learning about how the population perceived their health two years after the outbreak of COVID-19 will allow us to be more prepared and resilient in dealing with another future crisis.

### 1.1 Research question and hypotheses

Based on the associations found in previous studies and given the long-term presence of the virus in Israel, the study's research question of the relationship between anxiety and anhedonia symptoms with food consumption in the Israeli adult population since the outbreak of COVID-19 2020. In line with the research question, we hypothesize, in general, that there will be a relationship between anxiety and anhedonia. Anxiety and anhedonia will be associated with increased consumption of foods high in fat (specifically food with saturated fat) and sugar (when more than 15% of its weight is sugar). Specifically, participants reporting high anxiety levels and anhedonia will respectively report high consumption of fat and sugar intake. In addition, we hypothesize a relationship between anxiety and body weight. Further, an interaction between anxiety and body weight with increased consumption of high-fat and sugar foods will be observed. We expect that increased body weight participants reporting high anxiety will report the highest fat and sugar food intake.

## 2 Methods

This online cross-sectional study was conducted between February and May 2022 during the Omicron COVID-19 variant outbreak. The study was approved by the Ethics Committee of Tel**-**Hai Academic College (Ethical approval #: 11-3-2022), Northern District, 9977, Israel.

### 2.1 Participants

Participants were recruited through social media, with a general invitation to participate posted on WhatsApp, Facebook, and Instagram. Those who volunteered were assured of anonymity by separating personal details related to the participants from the collected data. Only the researchers had access to the obtained data. The questionnaires were then distributed on those digital platforms via a link sent to their phones. The study was administered to healthy Israeli residents aged 18 and above without chronic diseases, eating disorders, or under chronic pharmacological treatments. Participants not adhering to these inclusion criteria were rejected from participating in the study. Participation was possible only after signing an informed consent form. Participation was possible only after signing an informed consent form.

### 2.2 Study tools and data collection

We used the Qualtrics and Sona software for delivering the Beck Anxiety Inventory (BAI), the Snaith-Hamilton Pleasure Scale for Anhedonia Measurement (SHAPS), the Mediterranean Nutrition Questionnaire (I-MEDAS), and the demographic questionnaire. Participants, on a single occasion, answered questionnaires. All measurements concerned life routine in the last two years since the outbreak of the COVID-19 pandemic in 2020. Notably, the Israeli population was not locked down during this period.

#### 2.2.1 Beck Anxiety Inventory (BAI)

We used one of the most used versions of the questionnaire to evaluate anxiety symptoms. The Beck Questionnaire has been tested and validated in a wide range of studies over the years and found to be effective in assessing somatic and cognitive aspects of anxiety [17]. The instrument's reliability for the Israeli population has been evaluated with the sample of this study at Cronbach's Alpha = 0.933. The tool contains 21 questions ranging on a scale from 0 to 3 (0- “not at all,” 1- “slightly, I do not mind much,” 2- “moderately, it was very unpleasant, but I could bear it” and 3- “severely, I could hardly bear it”). Examples of statements from the questionnaire are: “fear that the worst will happen,” “frightened,” and “feeling of suffocation.” Each score was calculated on the scheme of 21 statements, a score ranging from 0 to 21 - low anxiety; 22–35 - moderate anxiety; 36 or higher – an alarming level of anxiety.

#### 2.2.2 Snaith-Hamilton Pleasure Scale (SHAPS) for anhedonia

The SHAPS questionnaire aimed to evaluate anhedonia, or the inability to experience pleasure. The questionnaire examined social interaction, consumption of food and beverages, sensory experience, and interest in leisure time. Reliability was measured at Cronbach's Alpha = 0.759. The questionnaire consists of 14 items, with four possible answers: strongly disagree, disagree, agree, and strongly agree. Everyone's score is calculated by summing up the 14 statements, where “disagree/disagree” answers will receive a score of 1**,** and strongly agree/agree answers will receive a score of 0. The meaning of the score: 2 or less - the norm range; more significant than 2 - abnormal range (the range is from 0 to 14) [18].

#### 2.2.3 The Mediterranean Nutrition Questionnaire (I-MEDAS)

The I-MEDAS questionnaire is a screening questionnaire adapted to Israeli food consumption according to the principles of the Mediterranean diet. A Mediterranean nutrition questionnaire was developed for Israelis in accordance with the original questionnaire developed in Spain [19]. The questionnaire includes 17 questions about how often certain foods are consumed. An example of the statement from the questionnaire: “How many legume dishes (such as lentils, white beans, chickpeas) do you eat per week? (1 serving = 120 g or 3/4 cup cooked legumes)”. A different scoring key for each question performs the calculation of the questionnaire. Based on the scoring key, a response that meets the required criterion receives 1 point**.** Any answer that differs from the required criterion earns 0 points. The final score will range from 0 to 17 points. A higher number of points correlates with better food consumption according to the principles of the Mediterranean diet. The instrument's psychometric properties in the Israeli population were evaluated with the sample of this study (Cronbach's Alpha = 0.655) [19].

#### 2.2.4 Body weight, serving size, and demographic variables

In addition to the Mediterranean nutrition questionnaire, the subjects were asked to answer questions concerning changes in their body weight and food portion sizes, all concerning the outbreak of the coronavirus pandemic and its effects. Finally, the subjects were asked to complete demographic details (such as age, origin, gender, and information about whether they were recovered/vaccinated – only if they wished; it was not necessary to answer these questions out of respect for the medical confidentiality of the subjects).

### 2.3 Statistical analysis

A priori power analysis was conducted using G*Power version 3.1.9.7 [20] to determine the minimum sample size required to test the study hypothesis. Results indicated the required sample size to achieve 80% power for detecting a medium effect, at a significance criterion of α = 0.05, was N = 280 MANOVA global effects analysis. Thus, the obtained sample size of N = 741 adequately tests the study hypothesis. The data was screened before analysis. Participants with any missing data or outliers observed by the researchers were rejected from the analysis. A descriptive statistic table was carried out to describe the demographic. Statistics were based on analysis of variance (ANOVA) and later examining various interactions and follow-up tests (The subsequent stages of the variance test allowed us to examine the shared and different effect of the variables). Finally, relationships between study variables (anhedonia, anxiety, weight, serving size, vaccines, isolations, and recovery) were measured using the Chi-squared test and Spearman's rank correlation coefficient. P values lower than 0.05 were considered statistically significant results. Confidence intervals were computed with 95%. All statistical analyses were done using the IBM SPSS statistic, version 27.

## 3 Results

**Fig. 1:**
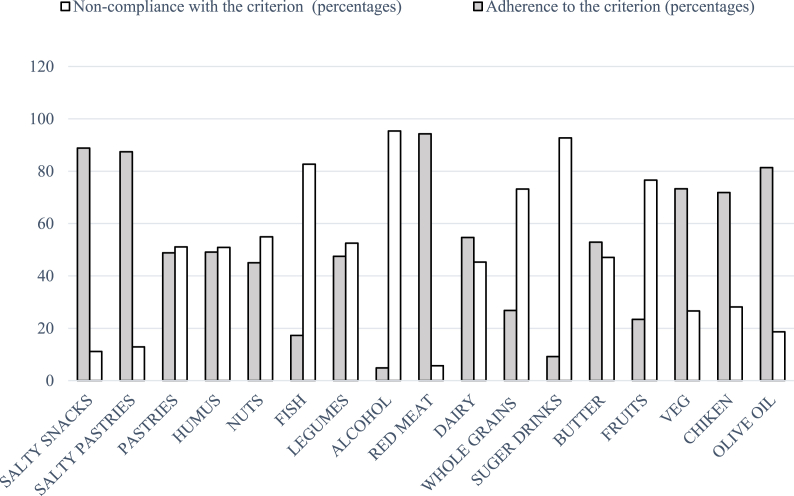
Daily/weekly average food consumption profile of the sample in the study presents the average food consumption profile of the sample in the study, according to the typical Mediterranean diet menu in Israel, with a percentage segmentation of the number of subjects who met the Mediterranean diet criteria and those who did not meet the criteria.

**Fig. 2:**
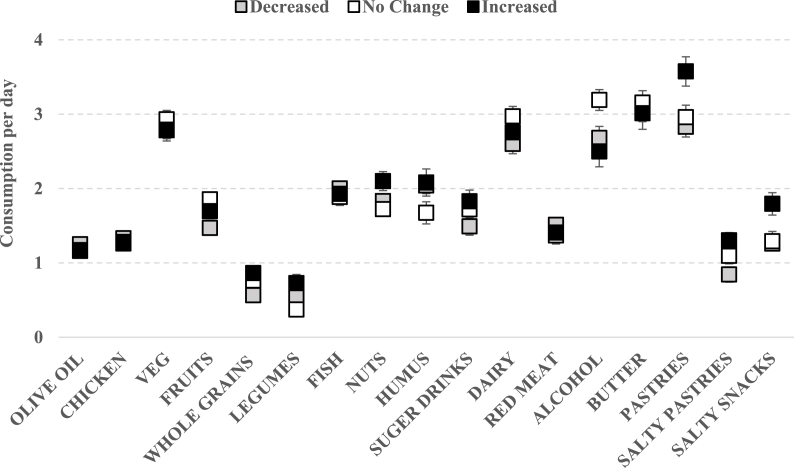
Food consumption by the change in weight depicts food consumption of each category examined in the study by the change in the participant's weight (no change, decreased, or increased).

Table 1 describes the distribution of this sample according to age groups, self-report of education level, self-report of changes in serving size and body weight, and reports such as isolation stay, COVID-19 infection**,** and vaccination. Of the 741 study participants, the average age was 35.56 ± 15.64, and 69.9% were female. Fig. 1 presents the average food consumption profile of the sample in the study, according to the typical Mediterranean diet menu in Israel, for subjects who met the Mediterranean diet criteria and those who did not. Fig. 2 depicts food consumption by the change in weight.

**Table 1:** Descriptive statistic.

	N	% of total	% Accumulated
*Age*			
18–29	394	57.4	57.4
30–59	224	32.6	90
60+	69	10	100
*Education*			
primary school	7	1.0	1.0
secondary school	284	41.3	42.4
B.A	280	40.8	83.1
M.A	104	15.1	98.3
PhD	12	1.7	100.0
*Serving size*			
no change	406	59.1	59.1
increased	172	25.0	84.1
decreased	109	15.9	100.0
*Body weight changes*			
no change	295	42.9	42.9
increased	253	36.8	79.8
decreased	139	20.2	100.0
*Quarantine*			
No	157	22.9	22.9
yes	318	46.4	69.2
more the once	207	30.2	99.4
Not interested in answering	4	0.6	100.0
*Infection with the virus*			
yes	337	49	49.05
No	344	50	99.12
Not interested in answering	6	0.87	100
*Vaccination against the virus*			
yes	635	92.43	6.84
No	47	6.8	99.27
Not interested in answering	5	0.72	100

Concerning anxiety, the average score was 11.719 (SD = 10.95). For anhedonia, the average score was 0.940 (SD = 1.705). Subjects with severe anxiety accounted for 5.1% of the sample, moderate anxiety about 10.9%, and low anxiety about 77.7%. Likewise, 11.2% of the subjects were anhedonic, and 88.8% were hedonic.

Several analyses were conducted further to elucidate the possible links between the examined variables: Table 2 presents the significant association between anxiety and anhedonia [χ^2^ = 37.868, p = .000]. Specifically, anhedonia was reported among the severe anxiety group. An additional association was observed between serving size and body weight [R_s_ = 0.428**, p = .001].

**Table 2:** Relationships between the variables using chi-square test and spearman correlation.

	anxiety	Anhedonia	weight	serving size
*Anxiety*	***	x [2] = 37.868, p = .000	Rs = 0.105**, p = .006	x [2] = 19.736, p = .000
*anhedonia*	x [2] = 37.868, p = .000	***	x [2] = 6.563, p = .038	s = 0.216**, p = .000
*weight*	Rs = 0.105**, p = .006	x [2] = 6.563, p = .038	***	Rs = 0.428**, p = .000
*serving size*	x [2] = 19.736, p = .000	s = 0.216**, p = .000	Rs = 0.428**, p = .000	***
*education*	Rs = −0.050, p = .192	x [2] = 5.426, p = .246	Rs = −0.119**, p = .002	Rs = −0.074, p = .051
*Infected with the virus*	Rs = −0.059, p = .123	x [2] = 14.380, p = .000	Rs = −0.035 p = .358	Rs = −0.060, p = .112
*quarantine*	Rs = .080*, p = .036	x [2] = 8.838, p = .032	Rs = 0.029, p = .441	Rs = 0.020 p = .602
*vaccination*	Rs = 0.057, p = .133	x [2] = 1.744, p = .418	Rs = 0.065, p = .087	Rs = 0.049 p = .201

MANOVA test revealed several essential aspects vis-à-vis weight variables, anxiety, and anhedonia. The interaction between weight, anxiety and anhedonia was significant [F (32, 2435.55) = 2.175, p = .000; Wilk's Λ = 0.901, partial η^2^ = 0.026]. Similarly, the interactions between weight and anhedonia and between weight and anxiety levels were significant [F (16, 1320) = 2.919, p = .000; Wilk's Λ = 0.933, partial η^2^ = 0.034, F (32, 2435.55) = 1.749, p = .006; Wilk's Λ = 0.920, partial η^2^ = 0.021], but not between anxiety levels and anhedonia [F (16, 1320) = 1.452, p = .110].

**Fig. 3:**
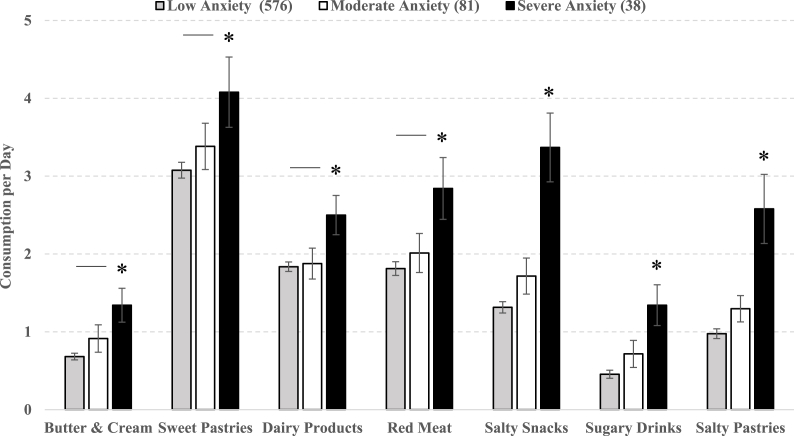
Food and drinks consumption based on the participant's anxiety levels depicts the statistically significant differences in food and drink consumption based on the participant's anxiety levels. Specifically, participants with severe anxiety levels consumed more butter and cream food, sweet pastries, dairy products, and red meat than participants with low anxiety levels. In addition, participants with both low and moderate anxiety levels consumed less salty snacks, sugary drinks, and salty pastries than those with severe anxiety levels – (*<0.05).

Fig. 3 depicts the statistically significant differences in food and drink consumption based on the participant's anxiety levels [F (16, 1370) = 4.296, p = .000; Wilk's Λ = 0.907, partial η^2^ = 0.048]. Further post hoc Bonferroni comparisons revealed that participants with severe anxiety levels consumed more butter and cream food (M = 1.342, SEM = 0.217) than participants with low anxiety levels (M = 0.682, SEM = 0.042). Similar results were found for sweet pastries (severe anxiety: M = 4.078, SEM = 0.451; low anxiety levels: M = 3.175, SEM = 0.436), for dairy products (severe anxiety: M = 2.500, SEM = 0.250; low anxiety levels: M = 1.836, SEM = 0.608), and for red meat consumption (severe anxiety: M = 2.842, SEM = 0.396; low anxiety levels: M = 1.812, SEM = 0.088). In addition, participants with both low and moderate anxiety levels consumed fewer salty snacks (low anxiety: M = 1.314, SEM = 0.072; moderate anxiety levels: M = 1.716, SEM = 0.231) than those with severe anxiety levels (M = 3.368, SEM = 0.442), less sugary drinks (low anxiety: M = 0.454, SEM = 0.051; moderate anxiety levels: M = 0.716, SEM = 0.174; severe anxiety levels: M = 1.342, SEM = 0.262), and less salty pastries (low anxiety: M = 0.975, SEM = 0.062; moderate anxiety levels: M = 1.296, SEM = 0.168; severe anxiety levels: M = 2.578, SEM = 0.444) – (p < .05).

**Fig. 4:**
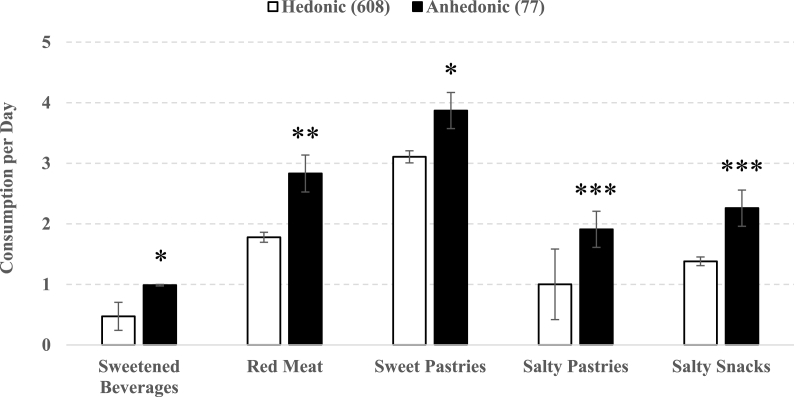
Food consumption based on hedonic/anhedonic participant's depicts the statistically significant differences in food consumption based on hedonic/anhedonic participants. Specifically, anhedonic participants consumed more sweetened beverages, red meat, sweet pastries, salty pastries, and salty snacks than hedonic participants (*<0.05, **<0.001, ***<0.000).

Fig. 4 depicts the statistically significant differences in food consumption based on hedonic/anhedonic participant's [F (5, 679) = 6.572, p = .000; Wilk's Λ = 0.954, partial η^2^ = 0.046]. Further post hoc comparisons using independent student t-tests revealed that anhedonic participants consumed more sweetened beverages (M = 0.987, SEM = 0.013) than hedonic participants (M = 0.472, SEM = 0.231), more red meat (anhedonic: M = 2.831, SEM = 0.305; hedonic: M = 1.778, SEM = 0.083), more sweet pastries (anhedonic: M = 3.870, SEM = 0.298; hedonic: M = 3.106, SEM = 0.100), more salty pastries (anhedonic: M = 1.909, SEM = 0.298; hedonic: M = 1.001, SEM = 0.582), and more salty snacks (anhedonic: M = 2.259, SEM = 0.299; hedonic: M = 1.381, SEM = 0.072) – [t (92.473) = −3.486, p = .017; t (87.814) = −3.329, p = .001; t (683) = −2.530, p = .012, t (683) = −4.622, p = .000; t (683) = −3.384, p = .000 – respectively].

**Fig. 5:**
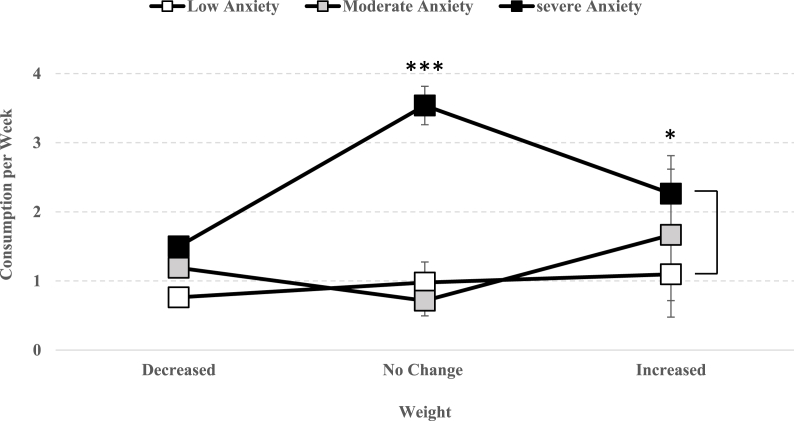
The interaction between anxiety and weight and the consumption of salty pastries depicts the interaction between anxiety and weight and the consumption of salty pastries. Specifically, among participants that gained weight, participants with severe anxiety levels consumed significantly more salty pastries than those with low anxiety levels. Similarly, among participants whose gain did not change, participants with severe anxiety levels consumed more salty pastries than those reporting low anxiety levels and those with moderate anxiety levels (N: low anxiety-decreased weight = 114, low anxiety-no change in weight = 265, low anxiety-increased weight = 197, moderate anxiety-decreased weight = 21, moderate anxiety-no change in weight = 21, moderate anxiety-increased weight = 39, severe anxiety-decreased weight = 6, severe anxiety-no change in weight = 13, severe anxiety-increased weight = 19; *<0.05, ***<0.000).

An analysis of variance was carried out to examine further the relationship between the various independent variables and the consumption of specific types of food. Fig. 5 depicts the interaction between anxiety and weight and the consumption of salty pastries [F (4, 686) = 2.993, p = .018, η^2^ = 0.017]. Post-hoc Bonferroni comparisons revealed that among participants that gained weight, participants with severe anxiety levels consumed significantly more salty pastries (M = 2.263, SEM = 0.550) than those with low anxiety levels (M = 1.096, SEM = 0.107) – [F (2, 252) = 5.833, p = .003, η^2^ = 0.044]. Similarly, among participants whose gain did not change, participants with severe anxiety levels consumed more salty pastries (M = 3.538, SEM = 0.951) than those reporting low anxiety levels (M = 0.977, SEM = 0.096) and those with moderate anxiety levels (M = 0.714, SEM = 0.219) – [F (2, 296) = 15.236, p = .000, η^2^ = 0.093].

**Fig. 6:**
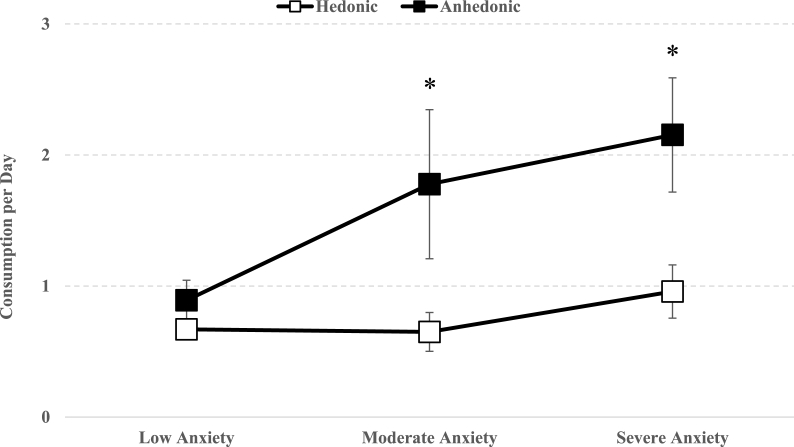
The interaction between anxiety, anhedonia, and butter and cream consumption depicts the significant interaction between anxiety, anhedonia, and butter and cream consumption. Specifically, among participants with moderate anxiety levels, anhedonic participants consumed significantly more butter and cream food than hedonic participants. Similarly, among participants with severe anxiety levels, anhedonic participants consumed significantly more butter and cream food than hedonic participants (N: low anxiety-hedonic = 524, low anxiety-anhedonic = 46, moderate anxiety-hedonic = 60, moderate anxiety-anhedonic = 18, severe anxiety-hedonic = 24, severe anxiety-anhedonic = 13; *<0.05).

Fig. 6 depicts the significant interaction between anxiety, anhedonia, and butter and cream consumption [F (2, 679) = 5.259, p = .005, ƞ^2^ = 0.015]. Post-hoc Bonferroni comparisons revealed that among participants with moderate anxiety levels, anhedonic participants consumed significantly more butter and cream food (M = 1.777, SEM = 0.569) than hedonic participants (M = 0.650, SEM = 0.148) - [F (1, 76) = 7.572, p = .007, η^2^ = 0.091]. Similarly, among participants with severe anxiety levels, anhedonic participants consumed significantly more butter and cream food (M = 2.153, SEM = 0.436) than hedonic participants (M = 0.958, SEM = 0.203) - [F (1, 37) = 8.012, p = .008, η^2^ = 0.186]. No differences were observed among participants with low anxiety levels [F (1, 568) = 1.995, p = .158].

**Fig. 7:**
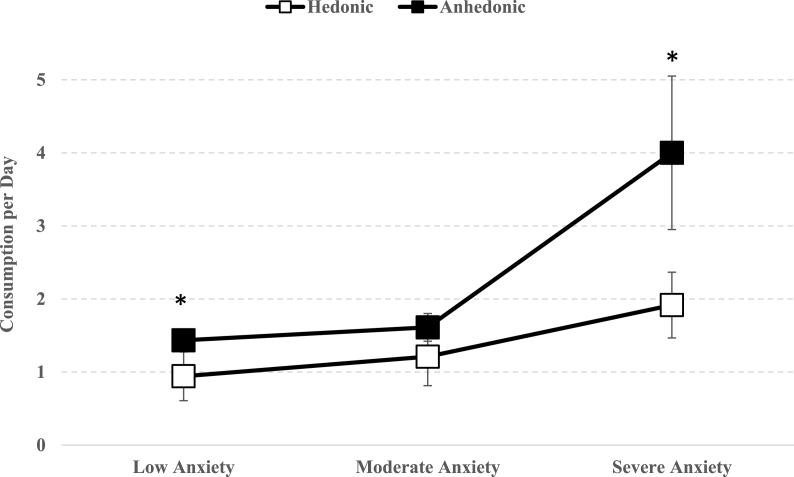
The interaction between anxiety, anhedonia, and salty pastries consumption depicts the significant interaction between anxiety, anhedonia, and salty pastries consumption. Specifically, among participants with low anxiety levels, anhedonic participants consumed significantly more salty pastries than hedonic participants. Similarly, among participants with severe anxiety levels, anhedonic participants consumed significantly saltier pastries than hedonic participants (N: low anxiety-hedonic = 524, low anxiety-anhedonic = 46, moderate anxiety-hedonic = 60, moderate anxiety-anhedonic = 18, severe anxiety-hedonic = 24, severe anxiety-anhedonic = 13; *<0.05).

Fig. 7 depicts the significant interaction between anxiety, anhedonia, and salty pastries consumption [F (2, 679) = 3.886, p = .021, ƞ^2^ = 0.011]. Post-hoc Bonferroni comparisons revealed that among participants with low anxiety levels, anhedonic participants consumed significantly more salty pastries (M = 1.434, SEM = 0.334) than hedonic participants (M = 0.942, SEM = 0.061) – [F (1, 568) = 4.580, p = .033, ƞ^2^ = 0.008]. Similarly, among participants with severe anxiety levels, anhedonic participants consumed significantly more salty pastries (M = 4.000, SEM = 1.050) than hedonic participants (M = 1.916, SEM = 0.334) – [F (1, 35) = 5.478, p = .025, ƞ^2^ = 0.135]. No differences were observed among participants with moderate anxiety levels [F (1, 76) = 0.928, p = .338].

Last, examining the possible effects of gender and age revealed additional associations. An association was found between anxiety and age; 22% of 18–29-year-olds reported moderate-severe anxiety compared to 5.7% of those aged 60 and over [x^2^ = 19.946, p = .001]. A significant correlation was found between age and weight; 38% of 18–29-year-olds reported weight gain compared with 28% of those aged 60 and over [x^2^ = 13.495, p = .009]. We found an association between gender and moderate-severe anxiety; 19% of the women reported moderate-severe anxiety compared to only 9% of men [x^2^ = 10.266, p = .036].

## 4 Discussion

This study examined the associations of psychological aspects such as anxiety and anhedonia on Israelis' nutritional habits and body weight changes after two years of the COVID-19 outbreak. The hypotheses were confirmed that differences would be found between anxiety levels and anhedonia, increased consumption of foods high in fat and sugar, and increased body weight. Those who reported severe anxiety and anhedonia also reported increased butter, snacks, and salty and sweet pastries consumption. They also reported the highest weight gain of all the study participants. To our best knowledge, an examination of the relationship between anxiety and anhedonia and their effects on the consumption of specific foods during the pandemic was found only in one other study conducted in Chile [15]. In line with the results presented here, their study found an association between severe anxiety and the consumption of foods high in fat and sugar, as well as an increase in body weight. Conversely, their study did not find a link between anhedonia and general food consumption.

Studies examining the level of anxiety in Israel before the pandemic outbreak found that 5.2% of Israeli citizens reported moderate and severe anxiety [21]. The participants in our study during the pandemic reported 16% moderate-severe anxiety. A meta-analysis of 17 studies on anxiety due to COVID-19 found that high anxiety levels were accompanied by additional mental effects such as anxiety, depression, and post-traumatic stress symptoms [4]. In addition, studies from Ireland, Hong Kong, and Italy also found increased anxiety due to the COVID-19 outbreak [14,22,23]. It should be noted that the level of anxiety found in our study among Israelis is lower than that found in the reports from other countries. These findings confirm our hypothesis regarding a relatively low level of anxiety among Israelis, probably due to the national resilience that characterizes Israelis [24]. Community resilience is another significant aspect that characterizes Israeli citizens during crises and/or disasters. It has been found that community resilience strengthens and improves the individual's personal and mental coping [25]. These anxiety data were consistent with the results of other Israeli studies during the COVID-19 period [24,26].

The associations presented here are consistent with other studies in Israel and worldwide during the pandemic. As in ours, other Israeli studies found a relationship between anxiety and weight gain. The percentage of those reporting weight gain in the study was 32.5%, while of those reporting mild and severe anxiety, 48% reported weight gain [24,26]. Studies from other countries, including Italy, Chile, the United States, Taiwan, China, and others, have also reported a link between anxiety and weight gain with the onset of the virus and in the long term [15,[27], [28], [29]]. The main findings of our study showed reports of increased consumption of carbohydrates, fats, and sugars. In a study conducted in Israel, 60% of the subjects claimed that their diet before the pandemic outbreak was healthier than during the pandemic, with 25% reporting weight gain and 10.7% moderate and severe anxiety [26]. In a study conducted in Italy, 46.1% reported increased consumption of sweet pastries and salty snacks, 19.5% reported weight gain, and 42.7% increased anxiety levels [30]. In an American study of 900 respondents, 42% reported increased snack consumption and anxiety regarding the pandemic [31].

Regarding anhedonia and food consumption during COVID-19, only one study reported an inverse and significant relationship between the quality and health of the respondents' diet and anhedonia [14]. The higher the level of anhedonia reported, the lower the quality and health of the diet [14]. Their data and ours are consistent with evidence that in situations involving anxiety, stress, and anhedonia, there is a risk of increased consumption of foods rich in carbohydrates, fats, and sugar and that this type of consumption can lead to weight gain [32]. People tend to regulate their emotions in situations that involve high anxiety or stress levels by consuming “comfort food” (fats, carbohydrates, and sugars) instead of choosing healthy food [33]. When we consume food, the level of dopamine in our brain increases, activating the reward and pleasure centers in the brain. Hence, people seek reward and satisfaction by consuming food despite lacking hunger signals, which Singh has termed “anhedonic hunger” [32].

Symptoms characteristic of high anxiety, anhedonia, and other psychological disorders are more common in those whose diet is poor and unhealthy [34]. A meta-analysis of 21 studies in different countries found that a Western diet characterized by increased consumption of red meat, sweets, snacks, saturated fatty foods, butter, and cream is associated with increased risk factors for anxiety and depression [35]. On the other hand, a Mediterranean diet characterized by higher consumption of fruits, vegetables, and low-fat and low-sugar products reduces the risk of anxiety [36]. Many of our bodily systems are involved when we consume food; among the effective systems is the intestine. The gut can affect mental health by communicating to the brain through the vagus neurotransmitters, regulating hormones, and influencing inflammation [37]. More specifically, the compositions of the microbial ecosystems of the intestines can regulate mental states and anxiety [38]. The microbiome-brain relationship is bidirectional since negative emotions can change the microbial ecosystem by releasing sympathetic stress hormone neurotransmitters [39]. Thus, diet and nutrition influence anxiety by regulating the microbiome. Studies on Western nutrition have found that it impairs and reduces the bacterial composition in the intestines and increases anxiety-inducing behaviors. In contrast, the Mediterranean diet reduces inflammation in the intestines and the risk of anxiety [17,40]. In our study, those who report the highest intake of fats and sugars also report the highest weight gain. A recent study reported that increased fat and sugar consumption carries significant risk factors, including obesity, diabetes, impaired immune system, and increased chances of intestinal inflammation [41]. Their study group, which increased its sugar and fat consumption, was at greater health risk for severe and life-threatening consequences of COVID-19 infection.

The results of our study on gender converge with the literature since our study found that women reported higher anxiety than men. Many studies have previously reached this conclusion in the presence of the pandemic and overwhelmingly regardless of the pandemic [42,43]. Another interesting aspect of our study was the difference between age groups in anxiety and weight reports, a difference that was found to be significant. We reported that the young adults had higher anxiety and weight gain levels -vs. adults aged 60 and over (22% vs 0.5.7%, and 38% vs. 28%, respectively). Similarly, a study conducted in the U.S. found that the level of anxiety in older adults was lower than that of young people and that the adults held a more optimistic and proportionate outlook [44].

### 4.1 Limitations and strengths

Among this study's disciplinary strengths were the sample size and diversity. The research questionnaire was published on several digital platforms, allowing for sample heterogeneity. There need to be more studies on the psychological aspects of the virus in Israeli lives, and this study expands knowledge in the field. This is the first study in Israel two years after the pandemic outbreak. It could contribute to more interdisciplinary work between nutritionists, psychologists, and psychiatrists. Also, this study provides a first insight into the potential influence of change on emotions and food consumption. However, more studies should examine the long-term effects of the virus on our lives from a psychological and nutritional perspective. The study must also improve, including reliance on self-reporting without clinical validation. Another weakness is the time the subjects were asked to respond; that is, they were asked to retrospectively examine two years of their lives. Although we asked the study participants to answer the questionnaires according to how the implications of COVID-19 affected them clearly and directly, some respondents noted that they had difficulty separating organic feelings of anxiety from daily life from those they felt were due to the pandemic per se. Another drawback was that despite the sample's heterogeneity, the number of participants aged 60 and above was limited, possibly because the study was delivered on a digital platform.

It should be noted that participants from the over-60 age group claimed that their diet was consistent and stable despite the pandemic since their health condition left them no choice. It is advised that future studies will focus on a more senior age group to encourage appropriate representation as part of the sample.

## 5 Conclusions

The current study emphasizes the far-reaching and long-term psychological effects of COVID-19 on the Israeli population. In a study examining the pandemic's long-term mental health, COVID-19 was defined as a multidimensional, integrated, and unique stressor [45]. This creates an urgent need to intervene and define new paradigms in mental health. As the pandemic lengthened, additional challenges were anticipated, shifting the initial focus of the virus outbreak from an immediate crisis to a long-term view, reflected in the professional discourse.

Taken together, the outbreak of the COVID-19 pandemic in Israel brought negative consequences, such as consuming foods high in fat and sugar and increasing body weight in those who reported high anxiety and anhedonia. More studies should examine the long-term effects of the virus on our lives from a psychological and nutritional perspective.

## Author contribution statement

Fleischer, E: Conceived and designed the experiments; Performed the experiments; Analyzed and interpreted the data; Wrote the paper. Landaeta-Díaz, L & González-Medinad, G: Conceived and designed the experiments; Wrote the paper. Horovitz, O: Conceived and designed the experiments; Analyzed and interpreted the data; Contributed reagents, materials, analysis tools or data; Wrote the paper.

## Data availability statement

Data will be made available on request.

## Funding sources

This research did not receive any specific grant from funding agencies in the public, commercial, or not-for-profit sectors**.**

## Declaration

The research has not been and will not be submitted simultaneously to another journal, in whole or in part.

The paper reports previously unpublished work.

All those named as authors have made a meaningful contribution to the research.

## Declaration of competing interest

The authors disclose no potential conflicts of interest.
