# Supplementary material for: Anxiety, anhedonia, and related food consumption in Israelis populations:An online cross-sectional study two years since the outbreak of COVID-19
Source: Heliyon. 2023 Jun 15;9(6):e17211. doi: 10.1016/j.heliyon.2023.e17211 (PMC10266889; doi:10.1016/j.heliyon.2023.e17211)
Supplement: Multimedia component 1 [file mmc1.docx]

**The interaction between anxiety and weight and the consumption of salty pastries**

| **Tests of Between-Subjects Effects** | | | | | |
| --- | --- | --- | --- | --- | --- |
| *Variable* | *F* | *Sig.* | *Partial Eta Squared* |  |  |
| Weight | 2.047 | 0.130 | 0.006 |  |  |
| Anxiety | 13.110 | 0.000 | 0.037 |  |  |
| Weight X Anxiety | 2.993 | 0.018 | 0.017 |  |  |
| **Multiple Comparisons** | | | | | |
| *Bonferroni* | | | | | |
|  |  |  | *Sig.* | *95% Confidence Interval* | |
|  |  |  |  | *Upper Bound* | *Lower Bound* |
| **No change** | *Minimal* | *Moderate* | 1.000 | -0.644 | 1.170 |
|  |  | *Severe* | 0.000 | -3.698 | -1.425 |
|  | *Moderate* | *Minimal* | 1.000 | -1.170 | 0.644 |
|  |  | *Severe* | 0.000 | -4.236 | -1.412 |
|  | *Severe* | *Minimal* | 0.000 | 1.425 | 3.698 |
|  |  | *Moderate* | 0.000 | 1.412 | 4.236 |
| **Increased** | *Minimal* | *Moderate* | 0.139 | -1.257 | 0.116 |
|  |  | *Severe* | 0.009 | -2.108 | -0.226 |
|  | *Moderate* | *Minimal* | 0.139 | -0.116 | 1.257 |
|  |  | *Severe* | 0.572 | -1.692 | 0.499 |
|  | *Severe* | *Minimal* | 0.009 | 0.226 | 2.108 |
|  |  | *Moderate* | 0.572 | -0.499 | 1.692 |
| **Decreased** | *Minimal* | *Moderate* | 0.478 | -1.159 | 0.305 |
|  |  | *Severe* | 0.507 | -2.028 | 0.555 |
|  | *Moderate* | *Minimal* | 0.478 | -0.305 | 1.159 |
|  |  | *Severe* | 1.000 | -1.737 | 1.118 |
|  | *Severe* | *Minimal* | 0.507 | -0.555 | 2.028 |
|  |  | *Moderate* | 1.000 | -1.118 | 1.737 |

**The interaction between anxiety, anhedonia, and butter and cream consumption**

| **Tests of Between-Subjects Effects** | | | | | |
| --- | --- | --- | --- | --- | --- |
| *Variable* | *F* | *Sig.* | *Partial Eta Squared* |  |  |
| Anhedonia | 25.011 | 7.265 | 0.035 |  |  |
| Anxiety | 8.673 | 0.0001 | 0.024 |  |  |
| Anhedonia X Anxiety | 5.258 | 0.005 | 0.015 |  |  |
| **Multiple Comparisons** | | | | | |
| *Bonferroni* | | | | | |
|  |  |  | *Sig.* | *95% Confidence Interval* | |
|  |  |  |  | *Upper Bound* | *Lower Bound* |
| **Hedonic** | *Minimal* | *Moderate* | 1.000 | -0.644 | 1.170 |
|  |  | *Severe* | 0.000 | -3.698 | -1.425 |
|  | *Moderate* | *Minimal* | 1.000 | -1.170 | 0.644 |
|  |  | *Severe* | 0.000 | -4.236 | -1.412 |
|  | *Severe* | *Minimal* | 0.000 | 1.425 | 3.698 |
|  |  | *Moderate* | 0.000 | 1.412 | 4.236 |
| **Anhedonic** | *Minimal* | *Moderate* | 0.139 | -1.257 | 0.116 |
|  |  | *Severe* | 0.009 | -2.108 | -0.226 |
|  | *Moderate* | *Minimal* | 0.139 | -0.116 | 1.257 |
|  |  | *Severe* | 0.572 | -1.692 | 0.499 |
|  | *Severe* | *Minimal* | 0.009 | 0.226 | 2.108 |
|  |  | *Moderate* | 0.572 | -0.499 | 1.692 |

**The interaction between anxiety, anhedonia, and salty pastries consumption**

| **Tests of Between-Subjects Effects** | | | | | |
| --- | --- | --- | --- | --- | --- |
| *Variable* | *F* | *Sig.* | *Partial Eta Squared* |  |  |
| Anhedonia | 16.622 | 0.001 | 0.023 |  |  |
| Anxiety | 17.838 | 2.814 | 0.049 |  |  |
| Anhedonia X Anxiety | 3.886 | 0.029 | 0.011 |  |  |
| **Multiple Comparisons** | | | | | |
| *Bonferroni* | | | | | |
|  |  |  | *Sig.* | *95% Confidence Interval* | |
|  |  |  |  | *Upper Bound* | *Lower Bound* |
| **Hedonic** | *Minimal* | *Moderate* | 0.477 | -0.7401 | 0.1923 |
|  |  | *Severe* | 0.003 | -1.6880 | -0.2599 |
|  | *Moderate* | *Minimal* | 0.477 | -0.1923 | 0.7401 |
|  |  | *Severe* | 0.127 | -1.5262 | 0.1262 |
|  | *Severe* | *Minimal* | 0.003 | 0.2599 | 1.6880 |
|  |  | *Moderate* | 0.127 | -0.1262 | 1.5262 |
| **Anhedonic** | *Minimal* | *Moderate* | 1.000 | -1.8587 | 1.5061 |
|  |  | *Severe* | 0.004 | -4.4660 | -0.6645 |
|  | *Moderate* | *Minimal* | 1.000 | -1.5061 | 1.8587 |
|  |  | *Severe* | 0.029 | -4.5914 | -0.1863 |
|  | *Severe* | *Minimal* | 0.004 | 0.6645 | 4.4660 |
|  |  | *Moderate* | 0.029 | 0.1863 | 4.5914 |
