# Supplementary material for: Anxiety, anhedonia, and related food consumption in Israelis populations:An online cross-sectional study two years since the outbreak of COVID-19
Source: Heliyon. 2023 Jun 15;9(6):e17211. doi: 10.1016/j.heliyon.2023.e17211 (PMC10266889; doi:10.1016/j.heliyon.2023.e17211)
Supplement: Multimedia component 2 [file mmc2.docx]

**Beck Anxiety Inventory (BAI)**

Below is a list of common symptoms of anxiety. Please carefully read each item in the list. Indicate how much you have been bothered by that symptom during the past month, including today, by circling the number in the corresponding space in the column next to each symptom.

|  | **Not at all** | **Mildly but it didn’t bother me much** | **Moderately – it wasn’t pleasant at times** | **Severely – it bothered me a lot** |
| --- | --- | --- | --- | --- |
| Numbness or tingling |  |  |  |  |
| Feeling hot |  |  |  |  |
| Wobbliness in legs |  |  |  |  |
| Unable to relax |  |  |  |  |
| Fear of worst happening |  |  |  |  |
| Dizzy or lightheaded |  |  |  |  |
| Heart pounding/racing |  |  |  |  |
| Unsteady |  |  |  |  |
| Terrified or afraid |  |  |  |  |
| Nervous |  |  |  |  |
| Feeling of choking |  |  |  |  |
| Hands trembling |  |  |  |  |
| Shaky / unsteady |  |  |  |  |
| Fear of losing control |  |  |  |  |
| Difficulty in breathing |  |  |  |  |
| Fear of dying |  |  |  |  |
| Scared |  |  |  |  |
| Indigestion |  |  |  |  |
| Faint/lightheaded |  |  |  |  |
| Face flushed |  |  |  |  |
| Hot/cold sweats |  |  |  |  |
