# Supplementary material for: Anxiety, anhedonia, and related food consumption in Israelis populations:An online cross-sectional study two years since the outbreak of COVID-19
Source: Heliyon. 2023 Jun 15;9(6):e17211. doi: 10.1016/j.heliyon.2023.e17211 (PMC10266889; doi:10.1016/j.heliyon.2023.e17211)
Supplement: Multimedia component 3 [file mmc3.docx]

**Data Dictionary**

| **Variable** | **Definition** | **Measurement's units** |
| --- | --- | --- |
| *Anxiety* | Excessive worry & apprehensive expectations about several events or activities *(DSM-V, 2013)*. | Beck Anxiety Inventory (BAI): scheme of 21 statements; a score ranging from 0-21 - low anxiety; 22-35 - moderate anxiety; 36 or higher – an alarming level of anxiety. |
| *Anhedonia* | Deficits in the capacity to feel pleasure and take interest in things *(DSM-V, 2013).* | Snaith-Hamilton Pleasure Scale (SHAPS) for anhedonia: 2 or less - the norm range; more significant than 2 - abnormal range (the range is from 0-14). |
| *Food consumption in Israel* | Mediterranean nutrition- the most common food consumption and diet recommendation in Israel. Mostly defined by low in saturated fat and high in vegetable oils consumption. | The Mediterranean Nutrition Questionnaire (I-MEDAS): score will range from 0 to 17 points. A higher number of points correlates with better food consumption according to the principles of the Mediterranean diet. |
| *Body Weight* | The mechanisms involved in body weight regulation in humans include genetic, physiological, and behavioral factors. Stability of body weight and body composition requires that energy intake matches energy expenditure and that nutrient balance is achieved ([Jéquier](https://journals.physiology.org/doi/full/10.1152/physrev.1999.79.2.451) and [Tappy](https://journals.physiology.org/doi/full/10.1152/physrev.1999.79.2.451),1999). | Self-reports concerning the outbreak of the coronavirus pandemic and its effects: no change; increased; decreased. |
| *Serving size* | The amount of food consumption. while constant increased consumption can lead to the development of obesity (Faulkner et al., 2012). | Self-reports concerning the outbreak of the coronavirus pandemic and its effects: no change; increased; decreased. |
