# Supplementary material for: Anxiety, anhedonia, and related food consumption in Israelis populations:An online cross-sectional study two years since the outbreak of COVID-19
Source: Heliyon. 2023 Jun 15;9(6):e17211. doi: 10.1016/j.heliyon.2023.e17211 (PMC10266889; doi:10.1016/j.heliyon.2023.e17211)
Supplement: Multimedia component 4 [file mmc4.docx]

**I-MEDAS**

| Foods and frequency of consumption | |
| --- | --- |
| 1 | Do you use olive oil as your primary source of oil? (in cooking and in addition to foods)  1. Yes; 2. No |
| 2 | Do you eat chicken, turkey, or meat **more often than** beef, hamburger, or sausage? (if vegetarian/vegan, mark "yes")  1. Yes; 2. No |
| 3 | How many servings of vegetables do you eat **a day**? (one portion = 200 gr. For example: large tomato, large pepper, 2 medium cucumbers; Adding vegetables to a main dish is considered 1/2 portion).  \|__\|.\|__\|__\| servings |
| 4 | How many servings of fruit do you eat a day? (Not including fruit juices) - (one serving= 125 gr. For example: medium apple, small orange).  \|__\|__\| servings |
| 5 | How many servings of butter, margarine, or sweet/cooking cream do you consume **per day**? (one portion = 12 grams, about 2 teaspoons).  \|__\|__\| servings |
| 6 | How many cups of sweetened drinks with sugar (such as: Fruits taste soft drinks, energy drinks, and non-diet Coca-Cola) do you drink **a day**?  \|__\|__\| cups |
| 7 | How many servings of whole grains do you eat **a day**? (such as: whole wheat bread or pasta, burghul, frike, buckwheat, grits, whole rice) - (one portion = a slice of bread or 1/2 cup of cooked cereal)  \|__\|__\| servings |
| 8 | How many servings of non-sugar-sweetened dairy products do you eat **a day**? (such as: milk, various types of cheese, yogurt, labneh) - (one portion = 1/2 cup of milk, a cup of yogurt, 150g of white cheese, labneh or cottage cheese, a slice of yellow cheese, about 75g of hard, salty cheese, a triangle of melted cheese)  \|__\|__\| servings |
| 9 | How many servings of red meat (such as: beef or lamb roast/steak), hamburger, or processed meat products (such as: sausage, hot dogs) do you eat **in a week**? (one portion = 150-100grams of red meat or 60grams of processed meat).  \|__\|__\| servings |
| 10 | How many servings of alcoholic beverages do you drink **a week**? (one portion = a cup of wine, a glass or can of beer, a small glass of a strong drink such as whiskey, vodka).  \|__\|__\| servings |
| 11 | How many legume dishes (such as: lentils, white beans, chickpeas) do you eat **a** **week**? (one portion = 150 grams or 3/4 cup of cooked legumes)  \|__\|__\| dishes |
| 12 | How many servings of fish do you eat **a week**? (One serving = 150-100 grams of fresh fish or canned tuna; 60 grams of salted/smoked fish).  \|__\|__\| servings |
| 13 | How many portions of nuts or peanuts (with or without salt) do you eat **per week**? (one portion = a handful, 30 grams)  \|__\|__\| servings |
| 14 | How many servings of hummus or tahini salad do you eat **a week**? (including tahini as addition to cooked foods, and salads containing hummus and tahini) - (one portion = table spoon).  \|__\|__\| servings |
| 15 | How many times **a week** do you eat sweet pastries (bought or home-made), such as: cakes, cookies, waffles, biscuits?  \|__\|__\| times |
| 16 | How many servings of salty pastries do you eat **a week**? (such as: burekas - filled pastry, Jahanon (traditional Yemenite dish), melawach (Yemenite pastry) - (one portion = 50-60 g, or 1 borax, 1/2 melawach, 1/2 jahanon)  \|__\|__\| servings |
| 17 | How many servings of salty snacks do you eat **a week**? (such as: peanut snack, Chips snack) - (one portion = 25g)  \|__\|__\| servings |
