# Supplementary material for: Anxiety, anhedonia, and related food consumption in Israelis populations:An online cross-sectional study two years since the outbreak of COVID-19
Source: Heliyon. 2023 Jun 15;9(6):e17211. doi: 10.1016/j.heliyon.2023.e17211 (PMC10266889; doi:10.1016/j.heliyon.2023.e17211)
Supplement: Multimedia component 5 [file mmc5.docx]

**Snaith-Hamilton Pleasure Scale (SHAPS)**

Rated on a 4-point Likert scale: 0 = strongly disagree, 1 = disagree, 2 = agree, 3 = strongly agree, except for items marked with *, which are reverse coded with answer choices as follows: definitely agree, agree, disagree, and strongly disagree.

1. I would enjoy my favorite television or radio program.
2. I would enjoy being with my family or close friends.
3. I would find pleasure in my hobbies and pastimes.
4. I would be able to enjoy my favorite meal.
5. I would enjoy a warm bath or refreshing shower.
6. I would find pleasure in the scent of flowers or the smell of a fresh sea breeze, or freshly baked bread.
7. I would enjoy looking smart when
8. I have made an effort with my appearance.
9. I would enjoy seeing other people's smiling faces.
10. I would enjoy a cup of tea or coffee or my favorite drink.
11. I would find pleasure in small things, e.g., a bright sunny day or a telephone call from a friend.
12. I would be able to enjoy a beautiful landscape or view.
13. I would get pleasure from helping others.
14. I would feel pleasure when I receive praise from other people.
